# Current Use of Cigarettes in the United States: The Joint Role of Race/Ethnicity and Health Insurance Coverage

**DOI:** 10.3390/healthcare11233014

**Published:** 2023-11-21

**Authors:** Julia N. Soulakova, Lisa J. Crockett

**Affiliations:** 1 Department of Population Health Sciences, College of Medicine, University of Central Florida, 6900 Lake Nona Blvd., Orlando, FL 32827, USA; 2 Department of Psychology, University of Nebraska-Lincoln, 315 Burnett Hall, Lincoln, NE 68588, USA; ecrockett1@unl.edu

**Keywords:** access to health care, communities of color, smoking cessation, tobacco treatment

## Abstract

The goal of this study was to assess the joint role of race/ethnicity and a health insurance coverage type (private, Medicare, Medicaid) in current cigarette use among adults in the U.S. Data from the 2019 Tobacco Use Supplement and the 2019 Annual Social and Economic Supplement of the Current Population Survey were merged (n = 39,882). Bivariate associations between each coverage type and smoking prevalence were examined within each of six racial/ethnic groups. A multiple logistic regression model (for the odds of current cigarette use) was estimated to explore the interactions between race/ethnicity and an indicator of each type of coverage among Hispanic, non-Hispanic (NH) Black/African American, and NH White adults. All analyses included survey weights. Results of bivariate analyses indicated that private and Medicare coverage were associated with significantly lower smoking prevalence (compared to no such coverage), while Medicaid coverage was associated with significantly higher smoking prevalence (all *p* ≤ 0.05). Some of these associations were significant among NH Black/African American and NH White adults (all *p* ≤ 0.05). The model indicated that the interaction between race/ethnicity and the indicator of private coverage was significant (*p* = 0.044): private coverage was significantly associated with lower prevalence among NH White adults only (AOR = 0.59, 98.3%, CI = 0.46:0.76). In addition, Medicaid coverage was significantly associated with higher smoking prevalence (overall). The study points to possible racial/ethnic disparities in the quality of smoking-related health care that people with the same type of coverage receive and possible underutilization of health care services even among adults with health insurance coverage, especially among communities of color and Medicaid enrollees.

## 1. Introduction

In this study we consider the joint role of race/ethnicity and health insurance coverage (private, Medicare, Medicaid) in the context of combustible-cigarette smoking in the U.S. While the prevalence of smoking decreased overall from 19.0% to 12.5% in the period from 2011 to 2020 [1], the reductions were not uniform across race/ethnicity [1]. Smoking prevalence remains highest among NH American Indian/Alaska Native adults and lowest among adults of Asian and Hispanic descent [2,3]. Therefore, it is important to continue smoking prevention and cessation efforts to reduce smoking prevalence in the general population and especially among communities of color with a higher prevalence of smoking and/or higher incidence of tobacco-attributed diseases relative to NH White adults [4,5,6,7,8].

Health insurance coverage plays an important role in smoking prevention and cessation in the U.S. [2,9]. According to the U.S. Census Bureau’s 2021 Report, the majority of people in the U.S. have private health insurance coverage [10]. In addition, there are several government programs (i.e., public health insurance coverage) including Medicare offered for 65+ year-old people and some people with long-term disabilities and Medicaid offered for people with low income [10]. Substantial efforts have been made to broaden health insurance coverage for smoking cessation in the U.S., particularly among Medicare and Medicaid recipients. For example, since 2011, healthcare providers have been reimbursed for providing cessation counseling for Medicare beneficiaries who smoke [11]. Moreover, the number of states offering comprehensive care for smoking cessation for Medicaid enrollees who smoke increased from six in 2008 to 15 in 2018 [12]. While health insurance coverage is associated with reduced smoking prevalence overall in the U.S. [2,9], there are disparities in smoking prevalence across different types of health insurance coverage as well as racial/ethnic disparities in health insurance coverage [12,13,14,15]. Specifically, smoking prevalence is highest among adults with Medicaid coverage and no coverage, and lowest among adults with private coverage [2,9]. Moreover, due to perceived discrimination and other barriers (e.g., lack of transportation) [16,17], some communities of color are more likely to lack access to or underutilize health care for smoking prevention and cessation relative to the other communities. For example, among adults who used cigarettes, the rates of receiving a doctor’s advice to quit smoking and using behavioral cessation interventions were consistently lower among Hispanic adults relative to NH Black/African American and NH White adults [18]. Therefore, it is important to assess the joint role of race/ethnicity and different types of health insurance coverage in the context of cigarette smoking. This knowledge would help determine if various types of health insurance coverage benefit adults from different racial/ethnic groups to the same degree or if there are racial/ethnic disparities in these benefits. Such disparities might reflect lack of access and underutilization of health care for smoking prevention and cessation by some underserved communities as well as reflect racial/ethnic disparities in the quality of health care people with the same type of coverage receive.

The goal of this study was to evaluate the racial/ethnic disparities in the associations between the type of coverage (private, Medicare, Medicaid) and current smoking among U.S. adults (18+ years old). To address this goal, we considered a number of factors that may influence smoking behaviors among adults: (1) sociodemographic characteristics including age, biological sex, marital status, highest level of education, annual family income, metro/non-metro area of residence, and U.S. region [3,9], (2) disability status [2,19], and (3) the TUS survey (administration) mode when examining smoking-related behaviors using the TUS data [20,21]. We hypothesized that after adjusting for these important factors, (H1) smoking prevalence would be lower overall among adults with private and Medicare coverage but higher overall among adults with Medicaid coverage, relative to adults with no such coverage, and (H2) the interactions between the race/ethnicity and each coverage type would be significant as the predictors of current cigarette use.

## 2. Materials and Methods

### 2.1. Data Source and Key Measures

We merged data from two Supplements of the Current Population Survey: the Tobacco Use Supplement (TUS, January and May 2019) and the 2019 Annual Social and Economic Supplement (ASEC). The TUS is a national survey of tobacco use among U.S. adults (18+ years old) [22]. The ASEC is a national survey of labor force and work experience (including income and noncash benefits) among 15+ year-old individuals in the U.S. [23]. The CPS is specifically designed so that the ASEC and TUS data can be linked [24]. To merge the TUS and ASEC data we used the linkage procedure previously described elsewhere [25]. The merged dataset included responses from 39,882 adults who self-identified as Hispanic, non-Hispanic (NH) American Indian/Alaska Native, NH Asian/Asian American, NH Black/African American, NH Hawaiian/Pacific Islander, and NH White, and for whom we had complete information on all study measures. Respondents who reported more than one race (i.e., multiracial) were not included in the study. The study dataset is available in the Harvard Dataverse repository [26].

The key measure of interest was an indicator of current cigarette use (yes, no) that was defined using TUS current smoking status (never, former, occasional, and daily). The indicator of current cigarette use differentiated between adults who reported occasional or daily use of cigarettes, and adults who reported never use or former use of cigarettes. The independent measures are listed in Table 1. The key independent measures included: (1) race/ethnicity (CPS), (2) the indicator of having private health insurance coverage (ASEC; yes, no), which differentiated between respondents who reported having current private coverage and those who reported not having private coverage, (3) the indicator of having Medicare coverage (ASEC; yes, no), which differentiated between respondents who reported having current Medicare coverage and those who reported not having Medicare coverage, and (4) the indicator of having Medicaid coverage (ASEC; yes, no), which differentiated between respondents who reported having current Medicaid coverage and those who reported not having Medicaid coverage. Note that respondents who reported having (or not having) a certain type of coverage may have had (or not) additional coverage type(s). Secondary independent measures included sociodemographic characteristics, disability status, and TUS survey mode (by phone, in-person). Disability status was defined using six ASEC items and differentiated between adults who reported having at least one of six disabilities (ambulatory, cognitive, independent living, hearing, self-care and vision) and those who reported having none of these disabilities.

### 2.2. Statistical Analyses

First, we computed descriptive statistics for the sample. Second, we estimated smoking prevalence for adults with and without each type of health insurance (private, Medicare, Medicaid) within each racial/ethnic group. Then, we examined bivariate associations between each type of health insurance and current cigarette use to assess the significance of the differences in smoking prevalence using Rao–Scott chi–squared tests [27]. In addition, we noted cases where cross-groups included 15 or fewer observations. These cases were excluded from further analysis. Finally, we performed a model-assisted analysis that incorporated a multiple logistic regression model predicting the odds of the current use of cigarettes. The initial model included three interactions: the interactions between the race/ethnicity and each coverage type (private, Medicare, Medicaid). The model controlled for all independent measures listed in Table 1. The final model was identified by excluding interaction terms (one at a time) with *p*-values exceeding 0.500 [removal of interaction terms with such *p*-values is not expected to affect the model fit and accuracy of classification]. All analyses included TUS survey weights and balanced repeated replications (with a Fay coefficient of 0.75) for variance estimation [25,28].

Analyses were conducted using SAS^®^9.4 package [29] as has been discussed elsewhere [30]. The study software codes are available in the Harvard Dataverse repositiory [26]. The significance level was fixed at the 5% level. Post hoc comparisons with Bonferroni adjustments were performed for significant interactions and significant main effects with multiple categories (three or more).

## 3. Results

### 3.1. Results of Bivariate Analyses

The overall smoking prevalence was 10.56% (SE = 0.40%). Table 1 presents the sample description in terms of independent measures. The overall proportion of adults with any insurance coverage (any private or public coverage, e.g., military health care) was 91.11% (SE = 0.40%). The overall proportions of adults who had a certain type of coverage (either alone or along with another type of coverage) were as follows: 69.20% (SE = 0.60%) of adults had private coverage (see Table 1), 34.36% (SE = 0.49%) of adults had public coverage (e.g., Medicare, Medicaid, military health care), 24.14% (SE = 0.28%) of adults had Medicare coverage (see Table 1), and 11.67% (SE = 0.44%) of adults had Medicaid coverage (see Table 1). Furthermore, 11.21% (SE = 0.29%) of adults had both private and Medicare coverage, 2.26% (SE = 0.17%) of adults had both Medicaid and Medicare coverage, and 1.09% (SE = 0.14%) of adults had both private and Medicaid coverage.

Table 2 presents the estimated smoking prevalence by health insurance coverage type for the sample as a whole and for each racial/ethnic group. First, Table 2 indicates that private coverage and Medicare coverage were associated with significantly lower smoking prevalence, while Medicaid coverage was associated with significantly higher smoking prevalence overall. Further investigation of the results presented in Table 2 indicated that the differences were significant only for some racial/ethnic groups: (1) among NH Black/African American and NH White adults, smoking prevalence was significantly lower for adults with private coverage relative to adults with no private coverage, (2) among NH White adults, smoking prevalence was significantly lower for adults with Medicare coverage relative to adults with no Medicare coverage, and (3) among NH Black/African American and NH White adults, smoking prevalence was significantly higher for adults with Medicaid coverage relative to adults with no Medicaid coverage. For the other racial/ethnic groups, while having private or Medicare coverage was associated with lower (or comparable) smoking prevalence and having Medicaid coverage was associated with higher smoking prevalence, the differences were not statistically significant.

Estimates for NH Hawaiian/Pacific Islander adults are not reported in Table 2 because sample sizes for some cross-groups were too small to conduct comparisons. Specifically, among NH Hawaiian/Pacific Islander adults only three adults with no private coverage, three adults with Medicare coverage, and one adult with Medicaid coverage reported current cigarette use. Furthermore, for the model-assisted analysis (described next) sample sizes for the cross-groups were sufficiently large only for the NH Black/African American, NH White, and Hispanic groups, so only those groups were considered.

### 3.2. Results of Model-Assisted Analysis Predicting the Odds of Current Cigarette Use

Table 3 presents the results based on the final model. The final model was significant (likelihood ratio CS = 16,011,132.6, df = 28, *p* < 0.001) and included two interaction terms. The interaction between the race/ethnicity and the indicator of having private coverage was significant (*p* = 0.044), while the interaction between the race/ethnicity and the indicator of having Medicare coverage was not significant. The interaction between race/ethnicity and the indicator of having Medicaid coverage was dropped during model building and thus was not included in the final model (see Table 3 footnote). Post hoc comparisons within each racial/ethnic group pointed to only one significant difference in smoking prevalence: among NH White adults, the odds of current cigarette use were lower for adults with private coverage relative to adults without private coverage (OR = 0.590, simultaneous 95% CI = 0.458:0.760). The corresponding odds ratios for Hispanic (OR = 1.349) and NH Black/African American (OR = 0.691) adults were not statistically significant.

The overall odds of current cigarette use were significantly higher for adults with Medicaid coverage relative to adults without Medicaid coverage (see Table 3). The overall odds ratio of current cigarette use was not significant when adults with Medicare coverage were compared to adults with no such coverage (see Table 3 footnote).

In addition, Table 3 indicates that the prevalence of smoking was higher among 25–44 and 45–64 year-old adults relative to 65+ year-old adults, among men relative to women, among married adults relative to adults who have been never married, among adults with lower levels of education relative to adults with a graduate (or equivalent) degree, and among adults with lower annual family incomes relative to adults with annual incomes of at least $75,000. In addition, the prevalence of smoking was significantly higher among adults with a disability relative to adults with no disability. Finally, in-person interviews were associated with lower smoking prevalence relative to the phone interviews.

## 4. Discussion

### 4.1. Key Findings

Our findings confirm that having private health insurance coverage is associated with reduced smoking prevalence overall in the U.S. However, the magnitude of the reduction in smoking prevalence differs by race/ethnicity. Smoking prevalence estimates unadjusted for other covariates indicated that among NH White and NH Black/African American adults (but not other racial/ethnic groups), having private coverage was significantly associated with lower smoking prevalence relative to not having such coverage. Moreover, the model-assisted results pointed to the same directional differences, but the association between private coverage and lower smoking prevalence was not significant among NH Black/African American adults (after adjusting for the other factors). Among Hispanic adults, the association between private coverage and lower smoking prevalence was not statistically significant in either the bivariate or model-assisted analyses.

The findings with respect to Medicare and Medicaid coverage are less consistent. While bivariate analyses indicated that Medicare coverage is associated with a significantly lower smoking prevalence overall and among NH White adults, model-assisted results pointed to no significant difference either overall or within any racial/ethnic group (Hispanic, NH Black/African American and NH White groups). Likewise, while bivariate analyses indicated that Medicaid coverage is associated with a significantly higher prevalence of smoking overall, among NH Black/African American adults, and among NH White adults, model-assisted results pointed only to a significant difference overall (i.e., Medicaid coverage was associated with a significantly higher prevalence of smoking overall). Therefore, we conclude that having private coverage is potentially more effective than Medicare coverage in terms of reduced smoking prevalence. In addition, having Medicaid coverage is potentially least effective among the three coverage types because Medicaid coverage is associated with higher smoking prevalence than no such coverage.

To summarize, our hypothesis (H1) that private coverage and Medicare coverage are associated with lower smoking prevalence, while Medicaid coverage is associated with higher smoking prevalence, was supported only with respect to private and Medicaid coverage (because after adjusting for other factors, the differences associated with Medicare coverage were not statistically significant). Our finding that Medicaid coverage is associated with higher smoking prevalence is consistent with prior literature [12,14,15]. In addition, our hypothesis (H2) that race/ethnicity would moderate the effects of each coverage type on current cigarette use was supported only with respect to private coverage. After adjusting for other factors, having private coverage was significantly associated with lower smoking prevalence only among NH White adults. This suggests that communities of color may benefit less than NH White adults from having private coverage.

The secondary findings, which concerned associations between sociodemographic characteristics and smoking prevalence, were consistent with those reported in the literature [3,9,31]. In addition, our finding that the prevalence of smoking was significantly higher among adults with a disability relative to adults with no disability was consistent with results from prior studies [19,32]. However, our finding that in-person interviews were associated with lower smoking prevalence relative to the phone interviews differed from results of a prior study suggesting that TUS interviews conducted in-person were associated with higher smoking prevalence compared to those conducted by phone in the period from 1992 to 2003 [21]. The discrepancy could be due to increased stigma associated with smoking making it harder for respondents to admit their smoking behaviors when they are interviewed in-person (relative to when they are interviewed by phone) [33,34].

### 4.2. Limitations

Our study has several limitations. First, the prevalence of smoking in our study (10.6% in 2019), is slightly lower than the prevalence computed using the 2018–19 TUS data (11.4%) [35]. We believe that the discrepancy is due to our use of the 2019 TUS which included data from only two survey waves, while the 2018–19 TUS data included data from all three survey waves. Including data from all three waves is expected to result in more accurate estimation of smoking prevalence in the U.S.

Second, while we attempted to include data for NH Hawaiian/Pacific Islander adults in this study, sample sizes were insufficient when health insurance coverage and smoking status were considered simultaneously. We encountered similar issues with respect to NH Asian/Asian American and NH American Indian/Native American groups and thus excluded these groups from the model-assisted analysis. Excluding these racial/ethnic groups is a common limitation in tobacco research [2,3,31]. Larger oversamples of these groups are needed to help inform efforts to improve access to and utilization of healthcare services among these communities.

Third, while in our pilot investigation we attempted to assess the significance of three-way interactions with race/ethnicity, e.g., by intersecting the race/ethnicity, the indicator of having private coverage and the indicator of having Medicaid coverage, we encountered some sample size limitations. For example, among Hispanic adults who had both, private and Medicaid coverage, only three adults reported current cigarette use.

Fourth, the study results may be due to confounding bias, e.g., among many other factors not considered in the study, sexual and gender minority status influences smoking behaviors [3]. Moreover, the study was observational and does not warrant any causal inferences.

Finally, we did not consider intersections among sociodemographic characteristics. Recent studies have discussed the importance of an intersectionality framework when estimating disparities in smoking-related behaviors [31,36]. For example, among intersectional groups (created via intersecting age, biological sex, race/ethnicity, and annual household income groups), smoking prevalence was highest among NH White men who were 35–54 years old and had low income [31]. Thus, we anticipate that differences in smoking prevalence associated with having (or not having) private health insurance coverage among NH White adults, could vary across diverse groups within the NH White population.

## 5. Conclusions

Substantial efforts have been made to broaden health insurance coverage for smoking cessation in the U.S., particularly among Medicare and Medicaid recipients. However, our study suggests that having private health insurance coverage has a more pronounced impact on smoking prevention and cessation than Medicare coverage and that Medicaid coverage is associated with higher smoking prevalence. Thus, in this regard, Medicaid remains the least beneficial option of the three types of coverage considered (private, Medicare, Medicaid). However, there could be racial/ethnic disparities in the benefits derived from having a certain type of health insurance coverage, e.g., in our study having private health insurance was associated with reduced smoking prevalence only among NH White adults (and not among people of color).

The study highlights the importance of informing the general public regarding the harmful effects of smoking (to help prevent cigarette use) and the availability of smoking cessation programs and medications (to enable successful cessation). It also underscores the need to remove the barriers to the utilization of health care for smoking cessation, especially among communities of color and Medicaid enrollees, and to reduce disparities in the quality of care that people of color and Medicaid enrollees receive. Furthermore, future tobacco research should include NH American Indian/Alaska Native and NH Hawaiian/Pacific Islander adults as well as other communities commonly underrepresented in tobacco research. Moreover, it is important to consider more in-depth intersections among sociodemographic characteristics and health insurance coverage types to identify subpopulations for whom smoking prevalence is especially high. However, considering the intersectionality among several factors would require considerably larger sample sizes for the corresponding cross-groups and thus may require revision of the existing national surveys of smoking behaviors or implementation of additional targeted surveys.

### Implications

Our study suggests that having private health insurance coverage may be more beneficial for smoking prevention and cessation than Medicare and Medicaid coverage. Nonetheless, non-Hispanic Black/African American and Hispanic adults with private coverage may benefit less in terms of reduced smoking prevalence than do NH White adults. Moreover, smoking prevalence remains higher among Medicaid enrollees relative to non-enrollees. The study highlights the importance of removing barriers to utilization of health care for smoking prevention and cessation, especially among communities of color and Medicaid enrollees.

## Figures and Tables

**Table 1:** Sample Description in Terms of Independent Measures (n = 39,882; N = 218,443,483).

Characteristic	Sample Count	Percent, % (SE, %) *
Age		
18–24	1928	10.83 (0.26)
25–44	12,447	33.62 (0.25)
45–64	13,730	33.62 (0.21)
65+	11,777	21.93 (0.15)
Sex		
Female	21,670	51.83 (0.24)
Male	18,212	48.17 (0.24)
Race/Ethnicity		
Hispanic	4330	16.45 (0.22)
Non-Hispanic (NH) American Indian/Alaska Native	354	0.76 (0.12)
NH Asian/Asian American	1698	6.07 (0.18)
NH Black/African American	3680	11.36 (0.25)
NH Hawaiian/Pacific Islander	103	0.27 (0.07)
NH White	29,717	65.09 (0.30)
Marital Status		
Married (living with a spouse)	21,644	51.40 (0.67)
Widowed, divorced or separated	10,078	21.24 (0.49)
Never married	8160	27.36 (0.53)
Highest Level of Education		
Less than high school	3416	9.95 (0.46)
High school or equivalent	10,492	25.71 (0.61)
Some college or bachelor’s degree	20,392	51.15 (0.64)
Graduate degree or equivalent	5582	13.19 (0.40)
Annual Family Income		
Less than $20,000	5414	12.83 (0.50)
$20,000–$39,999	8300	20.22 (0.61)
$40,000–$74,999	10,774	26.20 (0.75)
$75,000+	15,394	40.74 (0.85)
U.S. Region of Residence		
Northeast	6275	17.47 (0.24)
Midwest	8322	21.11 (0.22)
South	14,738	37.80 (0.32)
West	10,547	23.62 (0.28)
Metro/Non-metro Area of Residence		
Metropolitan area	31,276	86.62 (1.04)
Non-metropolitan area	8158	12.56 (1.03)
Unknown	448	0.82 (0.29)
Disability Status		
Yes	6098	12.49 (0.41)
No	33,784	87.51 (0.41)
TUS Survey Mode		
In-person interview	21,491	53.31 (0.78)
Phone interview	18,391	46.69 (0.78)
Indicator of Having Private Health Insurance Coverage		
Yes	27,549	69.20 (0.60)
No	12,333	30.80 (0.60)
Indicator of Having Medicare Coverage		
Yes	12,724	24.14 (0.28)
No	27,158	75.86 (0.28)
Indicator of Having Medicaid Coverage		
Yes	4119	11.67 (0.44)
No	35,763	88.33 (0.44)

* “SE” stands for “standard error”; percent is computed using the corresponding population count and the total population count (N).

**Table 2:** Smoking prevalence by type of health insurance coverage (private, Medicare, Medicaid) for diverse racial/ethnic groups (n = 39,882; N = 218,443,483).

Race/Ethnicity	Private Coverage			Medicare Coverage			Medicaid Coverage		
	Yes (%)	No (%)	*p*-Value*	Yes (%)	No (%)	*p*-Value*	Yes (%)	No (%)	*p*-Value*
Hispanic	6.02	7.21	NS	6.85	6.54	NS	8.50	6.11	NS
Non-Hispanic (NH) American Indian/Alaska Native	12.65	30.19	NS	18.17^a^	20.67	NS	27.12	18.58	NS
NH Asian/Asian American	4.01	3.55	NS	3.48^b^	3.96	NS	4.26^b^	3.83	NS
NH Black/African American	9.29	19.64	<0.001	13.57	13.54	NS	21.48	11.47	0.003
NH White	8.81	19.65	<0.001	9.58	12.32	0.002	27.58	10.14	<0.001
Overall	8.25	15.75	<0.001	9.54	10.89	0.050	19.64	9.36	<0.001

* NS stands for “not significant”. ^a^ Among NH American Indian/Alaska Native adults, only 13 adults with Medicare coverage reported current cigarette use. ^b^ Among NH Asian/Asian American adults only 15 adults with Medicare coverage and 9 adults with Medicaid coverage reported current cigarette use.

**Table 3:** Results based on the multiple logistic regression model for the odds of current cigarette use (n = 37,727; N = 202,937,900).

Measure*	Odds Ratio	Simultaneous 95% Confidence Interval**
Interaction Between Race/Ethnicity and Indicator of Having Private Coverage (*p* = 0.044)		
Hispanic: private coverage vs. no private coverage	1.349	0.624:2.917
NH Black/African American: private coverage vs. no private coverage	0.691	0.359:1.328
NH White: private coverage vs. no private coverage	0.590	**0.458:0.760**
Indicator of Having Medicaid Coverage (*p* = 0.048): yes vs. no	1.288	**1.002:1.657**
Age (*p* < 0.001)		
18–24 vs. 65+	0.848	0.391:1.841
25–44 vs. 65+	2.569	**1.561:4.228**
45–64 vs. 65+	2.766	**1.728:4.428**
Sex (*p* < 0.001): female vs. male	0.696	**0.600:0.809**
Marital Status (*p* < 0.001)		
Married vs. never married	0.648	**0.494:0.849**
Widowed, divorced or separated vs. never married	1.125	0.838:1.511
Highest Level of Education (*p* < 0.001)		
Less than high school vs. graduate degree (or equivalent)	4.823	**2.602:8.940**
High school or equivalent vs. graduate degree	4.426	**2.632:7.443**
Some college or bachelor’s degree vs. graduate degree	2.395	**1.440:3.982**
Annual Family Income (*p* < 0.001)		
Less than $20,000 vs. $75,000+	1.930	**1.313:2.836**
$20,000–$39,999 vs. $75,000+	1.759	**1.206:2.568**
$40,000–$74,999 vs. $75,000+	1.619	**1.140:2.299**
Disability Status (*p* = 0.020): yes vs. no	1.317	**1.045:1.659**
TUS Survey Mode (*p* = 0.018): in-person interview vs. phone interview	0.830	**0.712:0.968**

* The initial model contained three interactions. Because the interaction between the race/ethnicity and the indicator of having Medicaid coverage was not significant and had the largest *p*-value (*p* = 0.780), it was removed. The final model included two interactions, the interaction between the race/ethnicity and the indicator of having private coverage (*p* = 0.044) and the interaction between the race/ethnicity and the indicator of having Medicare coverage which was not significant (*p* = 0.275; detailed results are not presented). In addition to the main effects presented in the Table, the model included the following main effects: race/ethnicity (*p* < 0.001), the indicator of having a private coverage (*p* = 0.228), and the indicator of having Medicare coverage (*p* = 0.775), U.S. region of residence (*p* = 0.074) and metro/non-metro area of residence (*p* = 0.645). ** Statistically significant results are highlighted bold.

## Data Availability

The study dataset and SAS^®^ 9.4 software code can be found in the Harvard Dataverse repository [26].
